# Complete Genome Sequence of Hepatitis B Virus Genotype E: The First Molecular Characterization from an Imported Case in Mexico

**DOI:** 10.1128/genomeA.00187-16

**Published:** 2016-03-31

**Authors:** Noé Escobar-Escamilla, David Esaú Fragoso-Fonseca, Dulce María Arreguín-Porras, María del Carmen Esteban-Valencia, Estela Corona-Valdespino, Jaime Israel Falcón-Acosta, Roberto Vázquez-Campuzano, Fabiola Garcés-Ayala, Joanna María Ortiz-Alcantara, Irma López-Martinez, José Alberto Diaz-Quiñonez, José Ernesto Ramírez-González

**Affiliations:** a Instituto de Diagnóstico y Referencia Epidemiológicos (InDRE), “Dr. Manuel Martínez Báez,” Secretaría de Salud, Mexico City, Mexico; b Escuela Nacional de Ciencias Biológicas, IPN, Mexico City, Mexico; c Hospital General de México, Secretaría de Salud, Mexico City, Mexico; d Facultad de Medicina, UNAM, Mexico City, Mexico

## Abstract

Hepatitis B virus infection is currently a global public health problem. Here, we present the first characterization and complete genome sequence of a strain belonging to genotype E in Mexico, obtained from a foreign carrier with chronic infection.

## GENOME ANNOUNCEMENT

Hepatitis B virus (HBV) infection continues to be a public health problem worldwide, despite treatment and vaccination strategies. In 2010, 248 million individuals in the general population were chronically infected, and the estimated prevalence in Mexico is 0.19 to 0.21%, the lowest in the Americas region (1). HBV is the prototype member of the *Hepadnaviridae* family and it is classified into 10 genotypes (A to J), with different geographical distributions, clinical features, and responses to treatment (2). It has been suggested that the recently emergent HBV genotype E (HBV/E) originated in Nigeria and spread to West and Central Africa in the middle of the 20th century and became a hyperendemic strain in the region (3). Additionally, HBV/E is the more difficult genotype to treat with pegylated interferon–based therapy (4). HBV endemicity in Mexico is attributed to genotypes G and H (5), and identification of HBV/E has not been reported. Integral management of infected patients includes the monitoring of host and viral factors as well as different genotype responses to treatment (6). 

In a retrospective study on molecular characterization of different genotypes in a Mexican population (not published), we identified one sample belonging to HBV/E, which was obtained from an African traveler of temporary residence in 2011. Initially, DNA was extracted from plasma, and a fragment (429 bp) of the HBV S gene was amplified by PCR to perform direct Sanger sequencing. The identity of the partial sequence was established using the Genotyper tool at the International Repository for Hepatitis B Virus Strain Data (http://www.hepseq.org/Public/Web_Front/main.php) and deposited in GenBank (accession no. KP835522.1). Furthermore, the complete genome sequence was obtained through Ion Torrent technology. A single-end library was generated, resulting in 331,107 reads with an average length of 160 bp. The complete genome was assembled using TMAP version 4.2.18 with the sequence of HBV isolate PO04v2 (accession no. KF922439.1) as a mapping reference. The obtained contig had an average coverage of 129× and it was annotated and submitted using the NCBI BankIt tool. Phylogenetic analysis at the full-genome scale using 118 HBV/E sequences revealed that the identified strain is closely related to isolates from South Africa and Japan and belongs to the recently described Southwest African lineage (7). Additionally, the nonsynonymous A1762T and G1764A mutations in the basal core promoter region were found in the characterized strain. These nucleotide changes have been associated with increased viral replication levels (2) and proposed as predictive biomarkers for hepatocellular carcinoma development (8). 

Public policies for handling infected patients and disease control include treatment and prevention schedules, which are related to viral factors. For that reason, the surveillance of circulating HBV genotypes in the Mexican population and the screening of molecular markers associated with disease progression and prognosis will be helpful to understand the HBV molecular epidemiology and its relationship with the pattern of disease spread.

### Nucleotide sequence accession number.

The complete genome sequence of Hepatitis B virus strain InDRE 1109 has been deposited in GenBank under the accession number KT192626.
